# Can tape–screw fixation of a quadrupled semitendinosus graft in a full-length tibial tunnel provide superior fixation compared with a doubled semitendinosus–gracilis held with an interference screw? A matched-pair cadaveric biomechanical comparison

**DOI:** 10.1186/s10195-018-0495-x

**Published:** 2018-08-21

**Authors:** Christopher J. Vertullo, Joseph Cadman, Dané Dabirrahmani, Richard Appleyard

**Affiliations:** 1 Knee Research Australia, 8-10 Carrara St, Benowa, Gold Coast, QLD 4217 Australia; 2 Gold Coast Orthopaedic Research and Education Alliance, Griffith University, Gold Coast, QLD 4215 Australia; 3 Faculty of Medicine and Health, Macquarie University, Sydney, 2109 Australia

**Keywords:** Anterior cruciate ligament reconstruction, Biomechanical testing, Quadrupled semitendinosus graft

## Abstract

**Background:**

In anterior cruciate ligament reconstruction, quadrupled semitendinosus (Quad ST) grafts have potential advantages over doubled semitendinosus–gracilis (ST/G) including larger diameter and gracilis preservation, however the ideal tibial fixation method of the resultant shorter Quad ST graft remains elusive if a fixed-loop suspensory fixation device is used on the femur. We investigated whether the tibial fixation biomechanical properties of a Quad ST fixed indirectly with polyethylene terephthalate tape tied over a screw in a full outside-in created tunnel was superior to a ST/G graft fixed with an interference screw.

**Materials and methods:**

In a controlled laboratory study, six cadaveric matched pairs of each construct were subjected to cyclic loading to mimic physiologic loading during rehabilitation. This included preconditioning cycling, cyclic loading to 220 N for 500 cycles, then cyclic loading to 500 N for 500 cycles.

**Results:**

High standard deviations across the measured parameters occurred with no significant difference between measured parameters of elongation for the different constructs. Elongation of the Quad-ST construct was greater at 10 and 100 cycles, but not statistically different. Four of the six Quad-ST constructs failed below 100 cycles, compared with two failures below 100 cycles in the ST/G construct. There was a strong correlation between cycles to failure and bone mineral density for the Quad ST-tape constructs.

**Conclusions:**

Tibial fixation of Quad ST with a tied tape–screw construct in a full-length tunnel was not biomechanically superior to ST/G graft fixed with an interference screw, exhibited greater nonsignificant construct elongation with earlier failure, and was more reliant on bone mineral density.

**Level of evidence:**

In vitro laboratory study.

## Introduction

Currently, the ideal anterior cruciate ligament reconstruction (ACLR) graft and fixation choice remains elusive, with all common graft options having some disadvantages. Patella tendon autografts have become less popular in recent years [1], possibly due to concerns over donor-site pain and higher risk of osteoarthritis in cohort studies [2]. Allografts have documented higher revision rates [3, 4], however a great advantage is their absence of donor-site morbidity. Hamstring autografts have become more commonly utilized in recent years [1] in some regions, however concerns remain over higher failure rates in both registry studies [5] and randomized controlled trials [6] compared with autograft patella tendon grafts.

Gracilis harvest in addition to semitendinosus has been linked to knee flexor weakness by some authors [7, 8] and low rates of tendon regeneration [9], however the clinical relevance of this has been questioned by others [10].

Magnussen et al. [11] recently published data suggesting that smaller hamstring graft size was linked to higher failure in ACLR, and this has been confirmed by others [12]. While graft constructs of quadrupled semitendinosus are theoretically larger in diameter than doubled semitendinosus–gracilis (ST/G) grafts, reported matched-pair analysis to date surprisingly does not support this [13]. Quadrupled semitendinosus (Quad ST) grafts are also much shorter [14], making them unable to be fixed to the tibia with interference screws. While the shorter Quad ST grafts can be fixed with adjustable suspensory fixation devices on the tibia as well as the femur, concerns have been published over elongation of adjustable suspensory fixation devices under cyclic loading, with vigorous recent debate in literature [15–19]. In addition, tibial fixation in ACLR remains more problematic than femoral fixation, being described as the weak link in ACL fixation [20, 21]. Interference screw and polyethylene braided terephthalate (PET) tape fixation has been promoted as an alternative method for both tibial and femoral fixation of short quadrupled ST grafts, with supportive biomechanical data [14, 22, 23] for methods using partial length inside-out created tunnels, however this tape–screw method cannot be utilized with a fixed suspensory fixation loop on the femur.

The aim of this study is to investigate an alternative method of tape–screw construct fixation using full-length outside-in created tibial tunnels with tape tied over the screw for additional fixation. This alternative method would offer a graft tibial fixation option to surgeons who wished to preoperatively utilize a quadrupled ST graft to avoid harvesting the gracilis, or intraoperatively if a harvested gracilis tendon was inadequate in diameter or length to be used, while utilizing a fixed suspensory fixation device on the femur.

## Materials and methods

Two alternative ACLR tibial fixation methods were evaluated by matched-pair cadaveric laboratory biomechanical analysis: a ST/G graft with interference screw (ST/G-screw) and a Quad ST with 5-mm braided PET tape and screw (Quad ST-tape). Ethics approval was obtained from the institution’s Human Ethics Committee.

### Tibial preparation

Six matched pairs of seronegative cadaveric tibiae were thawed to room temperature and dissected (average age = 53 ± 5 years). All soft tissue was then removed. Then specimens were stored at room temperature prior to preparation, covered with gauze moistened with 0.9 % saline solution so as not to affect their mechanical properties [24]. The bone mineral density (BMD) of the tibia was measured using DEXA (Lunar Prodigy Advance Bone Densitometer, GE Healthcare), and they were then potted in poly(vinyl chloride) (PVC) pipe using polymethylmethacrylate. The matching semitendinosus and gracilis tendons were preserved for each tibia.

### Graft and tibial tunnel preparation

**Fig. 1:**
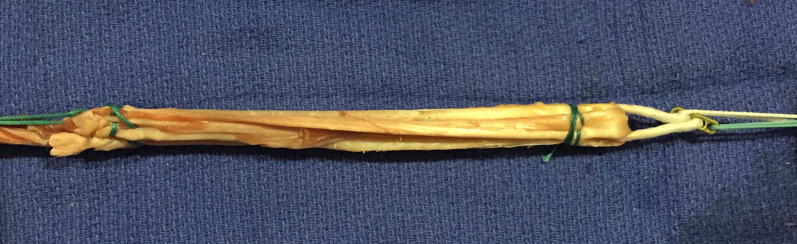
Number of cycles to failure for individual specimens

The tibiae were evenly assigned to each matched cadaveric hamstring ACL fixation graft construct. Each of the 12 graft preparations was performed by a 15-year postfellowship trained orthopedic surgeon (C.J.V.). Remnant muscle fibers were scraped from the tendons with scissors held at 90° to the tendon long axis. Six ST/G grafts were prepared using a Graftmaster III Graft Preparation System Board (Smith and Nephew, Memphis, TN). The ST/G tendons were proximally doubled over a 20-mm Endobutton CL, and the distal tendon ends were sutured with no. 5 Ethibond (Ethicon, Somerville, NJ, USA) in a locking pattern. A proximal #1 Ethibond (Ethicon, Somerville, NJ, USA) tripled circumferential suture was applied 15 mm distal to the proximal loop (Fig. 1).

**Fig. 2:**
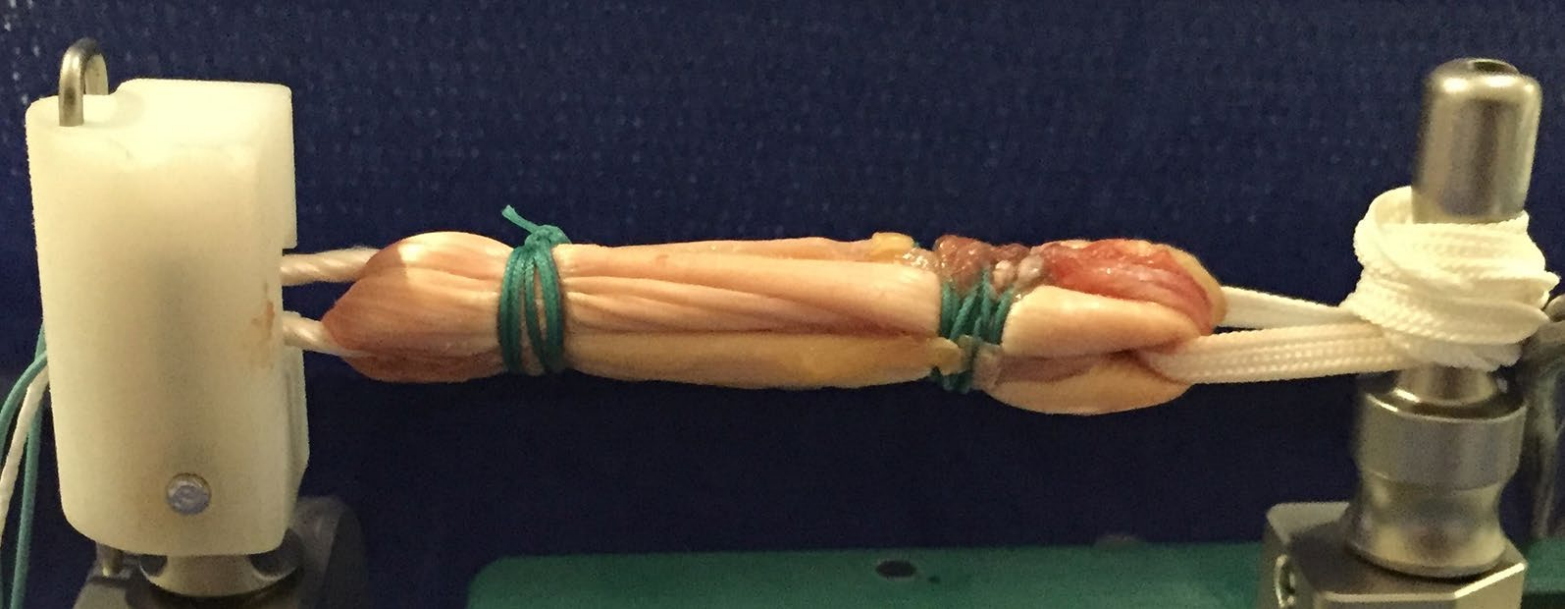
Prepared quadrupled semitendinosus graft undergoing pretensioning at 500 N

Six Quad ST-tape constructs were prepared using a modified Graftmaster III Graft Preparation System Board (Smith and Nephew, Memphis, TN) that could load the graft construct to 500 N. The Quad ST-tape construct preparation and suturing technique were the same on the tibial side as previously described for other Quad ST-tape techniques [14]. The ST tendons were quadrupled over a 20-mm Endobutton CL proximally and a 5-mm Endobutton PET tape loop distally (Smith and Nephew, Memphis, TN) (Fig. 2). An initial proximal and distal #1 Ethibond (Ethicon, Somerville, NJ, USA) horizontal suture was applied, then triple circumferential sutures were applied 15 mm distal to the proximal end and proximal to the distal end. The grafts were then tensioned at 500 N for 1 min.

The grafts were marked 50 mm from the proximal end, which corresponds to the 35-mm segment length of the articular ACL, plus 15 mm of femoral tunnel graft. All graft construct diameters and lengths were measured using an ACL Graft Sizer (Smith and Nephew, Memphis, TN).

The tibia ACL footprint was identified, and the planned intraarticular tunnel exit site was marked with a surgical marker, centered at the footprint of the native ACL. The distal tibial tunnel position was also marked, standardized at 6 cm distal to the tibial articular surface on the anteromedial tibial cortex. All tunnels were prepared using the Acufex ACL Aimer (Smith and Nephew, Memphis, TN). The tibial tunnel was drilled obliquely “outside-in” with a tibial tunnel guide set at 45° from the medial metaphysis into the anatomic footprint of the ACL. The planned length of the tunnels was 45 mm, and the diameter was a function of the measured graft diameter.

The grafts were drawn through the tibial tunnel, distally to proximally using traction on the Endobutton sutures, until 50 mm of the graft was above the proximal tibial tunnel aperture.

### Graft fixation

To fix the ST/G grafts to the tibiae, a Biosure interference screw in polyetheretherketone (PEEK) (Smith and Nephew, Memphis, TN) was inserted into the tibia tunnel parallel to the hamstring tendons over a guide wire with manual traction to the distal no. 5 Ethibond sutures. The screw size was based on the surgeon’s usual surgical technique strategy, matching the screw diameter to the graft and tunnel diameter, or downsizing the screw if the graft was between sizes, to maximum diameter of 8 mm. The 30-mm-long screws were inserted until there heads were flush with the tibial cortex.

The Biosure screw has a flat head, is slightly conical, and incorporates a consistent wall thickness throughout the length of the screw with a tapered body for easier insertion [25].

**Fig. 3:**
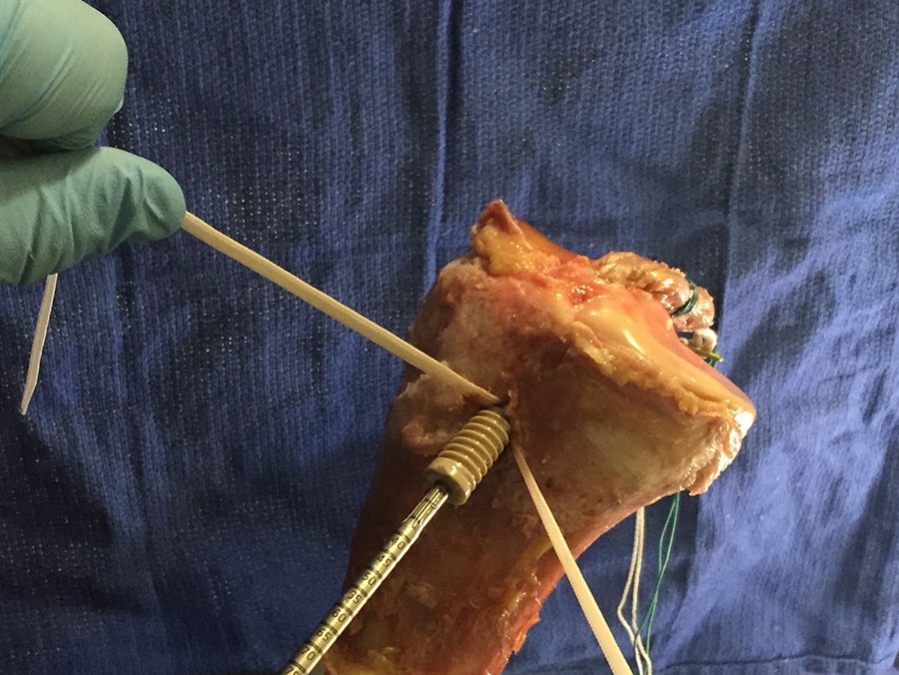
A PEEK screw is inserted with the tape under tension

In Quad ST-tape constructs, a 10- or 11-mm-diameter × 25-mm-length PEEK Biosure interference screw, oversized to tunnel size, was inserted into the tibial tunnel between the two tape distal ends. Manual traction was applied to the distal tape ends before and during screw insertion (Fig. 3). The screw was inserted until its head was flush with the tibial cortex. The tape was then tied over the screw heads with a surgeon’s knot [26], a variation of a square knot.

### Biomechanical tests

**Fig. 4:**
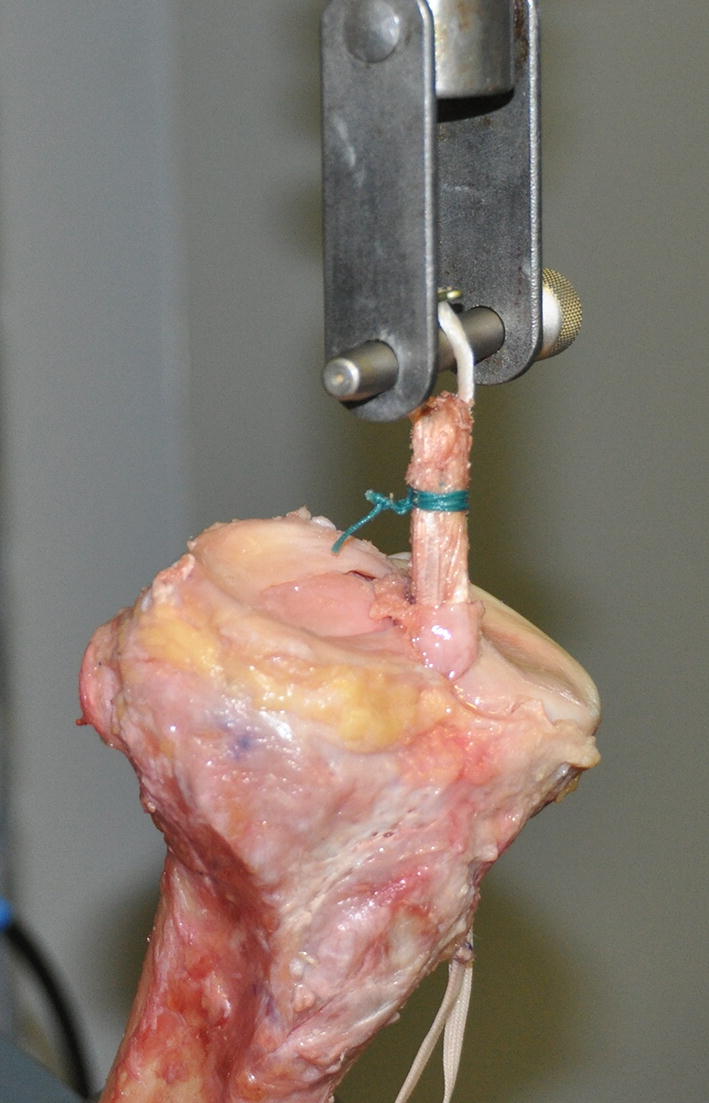
Quadruple semitendinosus tape construct in testing rig

Each tibia was mounted on the base of the testing machine (ElectroPuls E10000, Instron) so that the tibial tunnel was coaxial with the line of pull of the linear actuator (Fig. 4). The Endobutton 20 mm were looped around a steel cross-pin (5 mm diameter) simulating an identical femoral fixation for both constructs. Each construct was subjected to cyclic loading as follows: preconditioning cycling from 10 to 50 N for 10 cycles at frequency of 0.5 Hz, followed by cyclic loading from 50 to 220 N for 500 cycles at frequency of 1 Hz, followed by cyclic loading from 50 to 500 N for 500 cycles at frequency of 1 Hz. The aim of this protocol is to characterize the behavior of the construct during walking or other loading in patient’s activities of living, in which the applied strains are repetitive and similar [27].

**Fig. 5:**
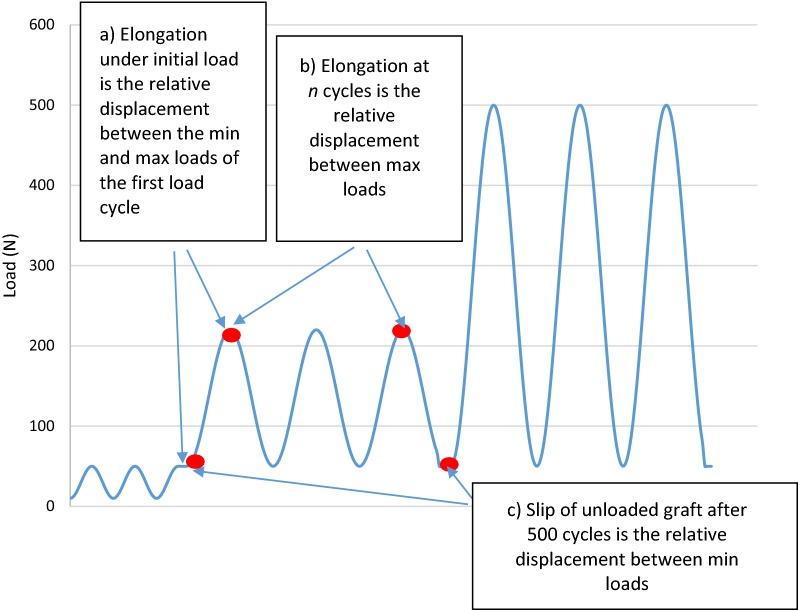
Schematic indicating where measurements were captured in the load cycle for (a) elongation under initial load, (b) elongation at *n* cycles, and (c) slip of unloaded graft after 500 cycles

Two high-definition cameras were set up orthogonal to the line of pull, and images captured at the following points: at 50 N prior to cyclic loading, at the peak of each loading cycle for the first 500 cycles at 220 N, at the 50 N load between the two cyclic loading regimes, at the peak of each loading cycle for the 500 cycles at 500 N, and at 50 N on completion of cyclic loading. The following results were captured: number of cycles to failure, elongation under initial load, elongation at 10 and 100 cycles, and slip of the unloaded graft after 500 cycles (Fig. 5). The mode of failure was also recorded for each test. Results were analyzed by two-tailed, paired *t*-test, where possible. When nonpaired comparison was undertaken, a standard *t*-test was utilized.

A custom image-processing program was developed in LabVIEW (National Instruments, Texas, USA) and used to calculate the displacements. The program allows the user to select common landmarks on bodies of interest and calculates the number of pixels between them. In this case, we selected a point in the graft construct and a point on the tibia. These raw data were scaled by finding the number of pixels over a known distance in the same images. In this case, the pin used to hold the femoral end of the construct was 8 mm, so it was used to calibrate the data. The displacement values described above were calculated by measuring the difference between the relevant readings.

### Statistical analysis and definitions

Failure of the construct was defined as relative displacement of the graft to the proximal end of the tibial tunnel exceeding 5 mm [28]. The elongation under initial load was defined as the relative displacement between the minimum load prior to cyclic loading (50 N) and the maximum load of the first cycle (220 N) (Fig. 5a). The elongation at *n* cycles was defined as the difference between the displacement at the maximum load of the *n*th cycle and the displacement at the maximum load of the first cycle (peak to peak) (Fig. 5b). The slip of the unloaded graft after 500 cycles was defined as the difference between the displacement at the minimum load cycle following 500 cycles and the minimum load prior to cyclic loading (Fig. 5c) (trough to trough). Note that these definitions only apply to cyclic loading with maximum load of 220 N, as most specimens did not survive loading at a maximum of 500 N.

Statistical analysis was undertaken using SPSS (IBM, New York, USA). Two-tailed paired Student *t*-tests were performed for measured parametric parameters with significance level set at 0.05, and Pearson’s correlation was used to analyze the BMD to number of cycles relationship.

## Results

Table 1 details the harvested tendon lengths, graft length, graft diameter, tibial drill diameter, and screw diameter. The Quad ST grafts had significantly larger mean diameter of 9.5 mm compared with the ST/G grafts (8.7 mm) (*p* = 0.0041).

**Table 1:** Diameter of quadrupled semitendinosus and doubled semitendinosus–gracilis

Specimen	Age (years)	Sex	Type of graft	Side	Diameter of graft (mm)	Lengths of harvested tendons (cm)	Length of graft (mm)	Tibial tunnel diameter (mm)	Tibial screw diameter (mm)
1	53	Male							
			Quad ST	Right	10	ST 31	60	10	11
			ST/G	Left	8.5	ST 32	120	8.5	8
						G 29			
2	52	Male							
			Quad ST	Right	10	ST 32	60	10	11
			ST/G	Left	9	ST 32	120	9	8
						G 24			
3	58	Female							
			Quad ST	Left	8.5	ST 25	60	8.5	10
			ST/G	Right	8	ST 27.5	110	8	8
						G 25.5			
4	54	Female							
			Quad ST	Right	9	ST 27	55	9	10
			ST/G	Left	8.5	ST 26	120	8.5	8
						G 24			
5	44	Male							
			Quad ST	Left	10	ST 32	60	10	11
			ST/G	Right	9.5	ST 30	130	9.5	9
						G 27			
6	55	Female							
			Quad ST	Left	9.5	ST 29	60	9.5	11
			ST/G	Right	8.5	ST 32	130	8.5	8
						G 31			

**Fig. 6:**
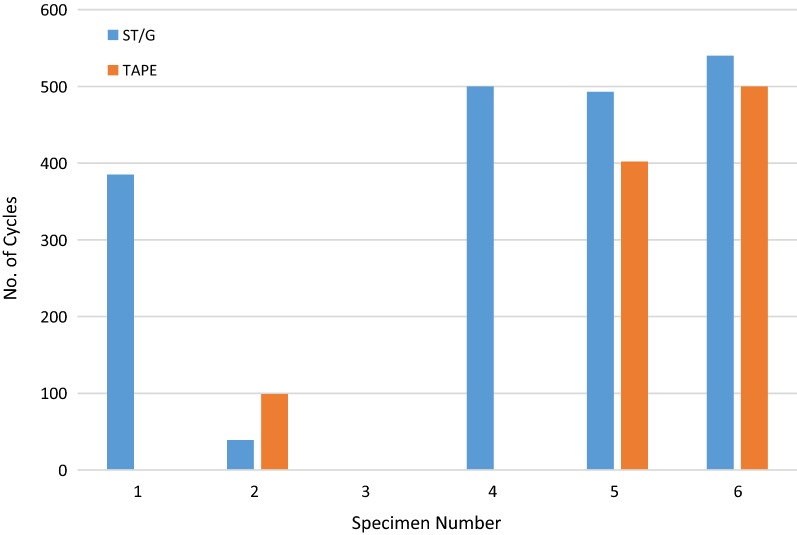
Number of cycles to failure for individual specimens (5 mm displacement)

All 12 constructs failed during cyclic testing, hence no load-to-failure tests were conducted. Eleven of the constructs failed during the first of the 500-N cycles, hence comparison of displacement results was only conducted for the first phase of cyclic loading, where the peak load was 220 N. The number of cycles to failure for each specimen is shown in Fig. 6. Two specimens survived more than 400 cycles at 220 N before failure, with failure defined as relative displacement between the graft and the tibial tunnel exceeding 5 mm. These two specimens (5 and 6 in Fig. 6) also had the highest bone mineral density (BMD) of all the tested specimen. Three specimens in the Quad-ST tape group and one specimen in the ST/G-screw group did not complete any cycles before the displacement exceeded 5 mm. As these four tests failed at 0 cycles, they do not appear in Fig. 6.

**Fig. 7:**
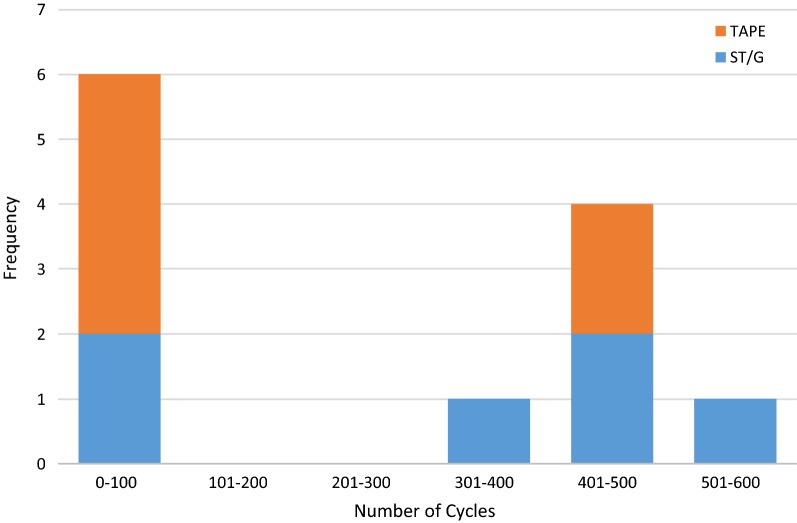
Histogram of number of cycles to failure for all specimens (5 mm displacement)

In the Quad ST-tape group, four out of six constructs failed below 100 cycles, and three of those failed in the first cycle. In contrast, the ST/G construct had two failures below 100 cycles, with the remainder of failures occurring after 385 cycles. The distribution of these failure data is presented in Fig. 7.

**Fig. 8:**
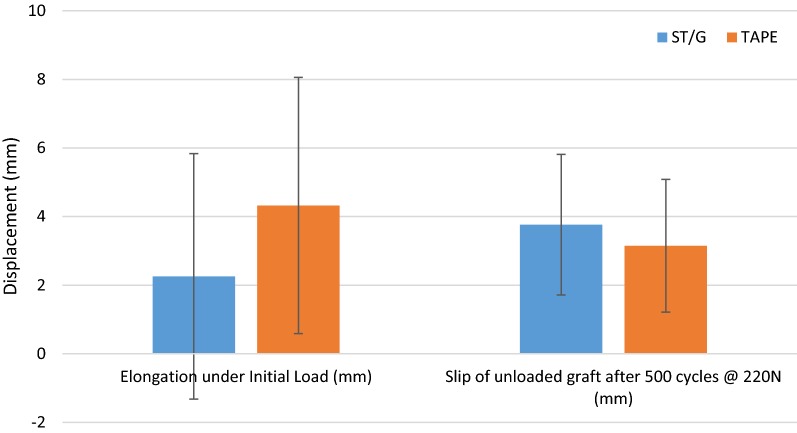
Results for elongation under initial load and slip of unloaded graft after 500 cycles

**Fig. 9:**
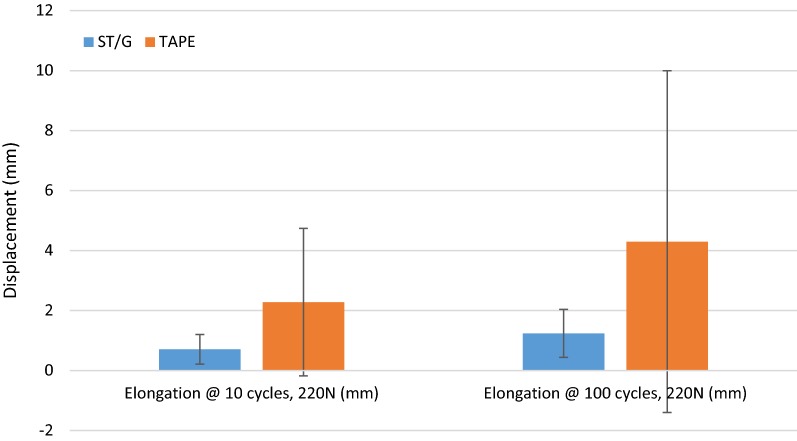
Tibial fixation elongation measured at 10 and 100 cycles

The displacement results for the ST/G and Quad ST-tape constructs, respectively, were as follows: elongation under initial load of 2.26 mm (SD 3.58 mm) and 4.32 mm (SD 3.73 mm) (Fig. 8), slip of unloaded graft after 500 cycles at 220 N of 3.77 mm (SD 2.05 mm) and 3.15 mm (SD 1.94 mm) (Fig. 3), and elongation at 10/100 cycles of 0.71 mm (SD 0.49 mm)/1.24 mm (SD 0.80 mm) and 2.28 mm (SD 2.46 mm)/4.3 mm (SD 5.70 mm) (Fig. 9).

**Fig. 10:**
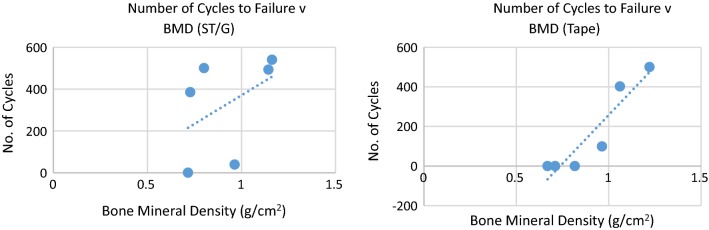
Relationship between number of cycles to failure and bone mineral density for semitendinosus–gracilis and quadrupled semitendinosus tape constructs

The average BMD for the ST/G and Quad-ST tape group was 0.92 and 0.91 g/cm^3^, respectively, similar to the higher BMD in younger adults, in whom this type of surgery is more common [20]. The number of cycles to failure and the displacement results showed strong or very strong correlation with BMD for the Quad ST-tape construct (*r* < −0.6, *r* > 0.9) (Fig. 10). The opposite was true for the ST/G construct (−0.5 < *r* < 0.3) (Table 2).

**Table 2:** Mode of catastrophic failure results

Mode of failure	Graft slipped around screw	Screw pulled through cortical bone	Cortical bone failed
ST/G	5		1
Quad ST		6	

The mode of failure for all specimens was displacement greater than 5 mm, with the modes of catastrophic failure for the two constructs being consistent (Table 3) but dissimilar. For the ST/G constructs, catastrophic failure was due to the graft slipping past the interference screw with the screw being left in place, except in one instance, where the cortical bone above the screw failed and the graft slipped out. For the Quad ST-tape construct, catastrophic failure occurred in five of the six constructs when the screw was pulled past the cortical bone enough for the knot to slip around the screw and pull through the cancellous bone. In one case the screw was pulled through to the tibial plateau by the knot.

**Table 3:** Correlation of measured variables versus bone mineral density

Description of correlation coefficient	ST/G	Quad ST
Number of cycles to failure versus bone mineral density	0.45	0.93
Elongation under initial load versus bone mineral density	−0.41	−0.80
Elongation at 10 cycles versus bone mineral density	−0.26	−0.62
Elongation at 100 cycles versus bone mineral density	0.05	−0.80

## Discussion

There were no statistically significant differences between the parameters measured for the two tibial fixation methods, however there were high standard deviations, a common observation in ACL fixation literature [14, 21, 23, 29]. When failure due to 5 mm of relative displacement is considered as the critical parameter, these large variations resulted in insufficient samples to accurately determine the significance of the result. The results are clustered into two groups, those that failed within 100 cycles at 220 N, and those that failed after 385 cycles at 220 N. A greater proportion of Quad ST-tape constructs failed earlier than ST/G constructs, which, while not statistically significant, raises concerns about the stiffness and fixation strength of the Quad ST-tape construct.

It is important to note that the image-processing method used cannot isolate the cause of the early displacement failures, hence it was not possible to ascertain whether this failure was due to initial laxity in the tape, a poor interface between the interference screw, tape, and bone, or the elasticity of the graft–tape interface.

The tunnel friction forces are difficult to define, as they depend on several factors including the normal force, the friction coefficient between the materials, and their contact area. Matching the tibial tunnel diameter to the ST/G diameter resulted in friction along the entire length of the graft–tunnel interface, playing a role in resisting the tensile loading for this graft compared with the shorter Quad ST-tape, which had much less graft tunnel contact. Friction along the graft–tunnel interface may have led to longer survival of two low BMD ST/G specimens compared with the matched pair with Quad-ST tape, suggesting that interference fixation would be preferable in patients with lower BMD.

The stability of the ST/G reconstruction also depends on the size and density of the tendons, with poor-quality tendons being more likely to deform plastically or slip past the screw [29]. It is possible that the early failure of a higher BMD specimen from the ST/G group was caused by such problems. In contrast, the tensile strength of the Quad ST-tape construct depends on the compression of the tape to the screw–bone interface [14, 22, 29] and the added restriction of the knot holding the tape in place around that screw [23], but not tunnel friction. One difference between the two groups is with regards to how the measured parameters correlate with the BMD. As noted in the results, BMD showed strong correlation with all results in the Quad-ST tape construct group (peak and residual displacements at 500 cycles are excluded because *n* = 2). In contrast, the same parameters showed weak correlation with BMD for the ST/G construct.

The difference in mode of catastrophic failure is also noteworthy. The ST/G reconstructions characteristically failed when the graft slipped around the interference screw; i.e., both the static friction along the graft–tunnel interface and the anchoring with the interference screw failed to counter the tension load. The majority of Quad ST-tape constructs failed when the knotted tape slipped around the screw into the cancellous bone. The interference screws used had a different design from other reports for a partial inside-out technique tape fixation technique, which may have resulted in the heterogeneous fixation results in our study. Full-length tunnels were used to mimic an ACLR technique using fixed suspensory loop femoral fixation with tibial tape–screw fixation. If our tunnels had been drilled partial length inside-out, our results may have been different; however, given that the majority of Quad ST-tape failures were due to graft slippage around the screw, despite the tape being tied over the screw head, it remains uncertain if the graft slippage still would not have occurred. If the tunnels had been drilled partial length inside-out so that an isthmus of bone remained at the screw tip, this may have provided additional friction to the tape. However, as noted above, we attempted to add to the tape–screw fixation by tying a knot over the screw in compensation for the full-length tunnel.

One other issue of concern is the tendency of the Quad ST-tape construct to fail by elongation earlier than the ST-G construct. While the sample size was not sufficient to clearly determine which failure results were outliers, elongation as a material property of the braided 5-mm PET tape we utilized has been reported by other authors in situations other than ACL fixation [30], but not described in studies using wider 7-mm PET tape for ACL fixation [14, 22, 29]. Why the braided 7-mm PET tape does not reportedly suffer elongation, while the braided 5-mm PET tape does, remains uncertain. In addition, our testing method could not differentiate elongation of the tape only, elongation of the Quad-ST graft in the tibial tunnel, or both.

One of the strengths of this study is the use of matched human tissue for both the bony and soft components. Magen et al. [31] suggested that animal tissue should not be used to estimate the performance of interference screw fixation in human tissue, and while Colderidge and Amis [27] used bovine tissue, they also stated that younger human cadaveric tibia would have been ideal. In an uncontrolled 7-mm tape and 10-mm screw biomechanical study, Collette et al. [14] utilized cadaveric tissue; however, they used femoral heads, rather than cadaveric tibia, which would have had very different bone density and corticocancellous structure from a tibia. The only other study to use cadaveric tibia in a tape and screw study to date is that of Birmingham et al. [23], who reported mean failure load of 136 N (±136 N) with a 10-mm screw, compared with 288 N (±77 N) for the comparator Endobutton group. When they used a larger 12-mm screw and tied the tape over an additional button, they achieved a statistically significantly greater mean failure load of 668 N (±278 N), with the additional cortical fixation. It should be noted that we tied the tape over an 11-mm screw, rather than to an additional button, hence we did not gain cortical fixation. They reported greater stiffness in the larger screw–button–tape group than the standard screw–tape group and comparator Endobutton group, but the same migration.

The main weakness of the study is the limited number of matched constructs, which when combined with the high variability of measured parameters resulted in insufficient samples to accurately determine the significance of the results when failure due to 5 mm of relative displacement was considered as the critical parameter.

## Conclusions

Tibial fixation of ACL quadrupled ST grafts tied over a tape–screw construct with a full outside-in created tunnel was not superior to ST/G grafts fixed with an interference screw.
